# Trajectories of physicians in Manitoba, Canada: the influence of contact points of rural-focused professional learning

**Published:** 2018-11-12

**Authors:** John Murray, Charles Penner, Wayne Heide, Dawn Piasta, Don Klassen

**Affiliations:** 1 Department of Family Medicine, Max Rady College of Medicine, Faculty of Health Sciences, University of Manitoba, Manitoba, Canada; 2 Department of Curriculum and Pedagogy, Faculty of Education, Brandon University, Manitoba, Canada; 3 Manitoba Office of Rural and Northern Health, Dauphin, Manitoba, Canada

## Abstract

**Background:**

The Manitoba Office of Rural and Northern Health (ORNH) provided a multi-year series of elective opportunities for undergraduate medical students to support rural/remote medical practice. The purpose of this study was to examine the career trajectories of Manitoba physicians in eight matched cohorts over the period 2004-2007 between: 1) those who experienced a required rural clinical block rotation only during their undergraduate medicine training in Manitoba (Med 1 and Med 3), and; 2) those who engaged in and completed additional elective programs referred to here as “contact points”.

**Methods:**

The study utilized a retrospective/longitudinal matched cohort design which included the common factor of a mandated rural clinical one-week rotation and the differentiating factors of experiences in elective programming offered by the ORNH (contact points).

**Results:**

Of the 344 Manitoba-trained physicians whose location of current practice could be determined, 74 are presently in rural/remote communities and 270 in urban settings. Those physicians who are now in rural/remote practice were significantly more likely (*p ≤ 0.05*) to have continued contact with ORNH in addition to the mandatory rural rotation alone. For practitioners now located in rural/remote settings, a mean of 0.903 contact points per learner with ORNH programs is observed. For those now in urban practice the mean number of contact points per learner was 0.233.

**Conclusion:**

We conclude that there is an association between rural-focused contact points and rural and remote practice in Manitoba. Targeted professional learning where physician recruitment and retention remains a continuing challenge is discussed.

## Introduction

Regional disparity in the recruitment and retention of physicians, and the geographic and demographic maldistribution of physicians in Canada is an enduring complex problem which has generated a substantial literature in the last two decades.^1-9^ The problem comprises many factors which are entwined in a complex interplay that continues to present both research design difficulties to examine the problem and a stubborn defiance for the development of effective strategic solutions in the wake of the research evidence.^3, 10-15^

If an assumption is made that context-dependency is an important factor in teasing out the issues related to the maldistribution component of the problem, then the unique identifiers within a Canadian province present opportunities for place-based research as opposed to the barriers of seeking after a broader, national consensus model.^16-23^ This context-dependency is often illustrated in the literature as inter-comparisons among three federal democracies: Australia, Canada, and the United States.^24-26^ In keeping with the traditions of seeking after research-based, evidential arguments to bring scope to the problem, the notion of the “innovative intervention” and *post-hoc* analysis and recommendations has become somewhat standard within the medical education research community.^11,13,20-21,27-29^ Such interventions are occurring as (or in): (a) incentives for the establishment of smaller, geographically responsive and context-dependent regional medical campuses; (b) satellite medical campuses such the program established at Brandon, Manitoba, and; (c) a variety of distributed medical education (DME) models. Many workers are examining more closely the sociological determinants of medical student and practitioner choice as to eventual location of practice.^6,10,16,20,30,32-38^ Additionally, certain countries facing the maldistribution problem are now actively establishing the longitudinal integrated clerkships (LICs) as an innovation of contrast to the traditional four- to six-week block rotations. Fortunately, LICs are also providing the experimental basis for possible solutions to the rural placement difficulties by virtue of matched-cohort studies and their associated research outcomes.^39-45,47^ For instance, researchers have developed a typology of LICs for Australia.^46^ Given that both Canada and Australia have a first-order similarity in their respective healthcare systems, the Australian LIC model could potentially transfer well to Canada.^9,41,42,46^ New efforts by the DME Resource Group of the Association of Faculties of Medicine in Canada (AFMC) are seeking the development of regional typologies and a terminological framework for DME. This study takes a longitudinal view to assess the impact of an extended series of co- and extra-curricular programs specific to encouraging rural general practice among physicians in the Province of Manitoba. Therefore, it can be classed as context-dependent, regional in scope, and site-specific.^1,4,11,25,31,40,48^ This study contributes to a growing number of matched-cohort research designs specific to addressing rural and remote issues of physician supply and retention.^6,22,44,49^

The Manitoba Office of Rural and Northern Health (ORNH), since 2003, has provided a multi-year series of elective opportunities for undergraduate medical students and those in practice to support rural/remote medicine with a particular reference to primary care and specialization at the local level. The purpose of the study was to examine the career trajectories of Manitoba physicians in four matched cohorts over the period 2004-2005 to 2007-2008 with the following distinctions: 1) those who experienced a mandatory rural clinical block rotation only during their undergraduate medicine training in Manitoba (at Med 1) and a mandatory clerkship rotation (in Med 3), and; 2) those who engaged in and completed additional elective programs offered through the ORNH. We refer to the elective experiences in (2) as “contact points.”

### Background

#### The Office of Rural and Northern Health

Manitoba’s Office of Rural and Northern Health was established in 2002 to address long-term recruitment and retention issues in rural and northern Manitoba across all healthcare professions. Synergistically, the mandate of the ONRH was to address the stubborn problems of regional disparity and maldistribution of physicians working in primary care through strategic research-based solutions.

The ORNH implements initiatives at all phases of the training cycle with a view to encouraging rural and northern situated individuals to consider careers in health care; complete some of their training in rural, northern or remote sites, and; ultimately choose to work and live in rural and northern Manitoba environs.

Perhaps the most visible initiative of the ORNH began in 2003 – the Rural Week experience.^a^ Initially, Rural Week was an elective program for first-year medical students in the Faculty of Medicine at the University of Manitoba, providing an *in situ* opportunity to capture both insights into community-based rural and northern medical practice and to have the communities demonstrate to Year 1 students what the rural northern lifestyle had to offer.

The objectives of the Rural Week experience, included the following: 1) develop a basic understanding of the family physician role within a rural/remote community setting whether this be in the outpatient office, in-patient care, emergency room coverage, or obstetrical care where applicable; 2) identify the unique nature of the demands on a rural physician as part of a multi-disciplinary health care team; 3) develop understanding of other health care team members’ roles; 4) assess patients under the supervision of the local family physician, and; 5) appreciate the lifestyle of the present-day rural/remote physician. All principal objectives and expectations were intended to be met through direct and daily contact with preceptors in addition to making connections among nurses, pharmacists, and an extensive network of inter-professional health care workers.

In addition to the Med 1 Rural Week program, students and post-graduates were provided a variety of co- and extra-curricular opportunities developed by the ORNH. Some examples of these activities provided to physicians-in-training and practitioners by the ORNH include (details of these can be accessed online at the links provided):

- Rural Week ^a^
- Home for the Summer Program^b^
- Summer Work, Education, and Training (SWEAT)^c^
- Rural Manitoba Health Mentorship Program (RMHMP)^d^
- Manitoba Medical Students’ Rural Interest Group^e^
- University of Manitoba Family Medicine Residents’ Retreat

## Methods

### Design

The study utilized a retrospective/longitudinal matched cohorts design comparing the two contact points of a mandated rural clinical block rotation and the differentiating factors of experiences in elective programming offered by the ORNH. Location of current rural/remote practice provided the subsequent outcome following a minimum latency period of nine years and for as long as twelve years to 2016-2017.

For our purposes, the focus of this study was the programming offered by the ORNH outside of the mandated Rural Week and the rural family medicine block during clerkship. We consider these elective experiences as the equivalent of voluntary interventions, a time series of professional learning opportunities, or “points of contact.” What became attractive to us, given the compulsory nature of Rural Week in the Med 1 curriculum and the mandatory clerkship rotation in rural family medicine at Med 3, was to select a series of four matched cohorts from the academic years 2004-2005 to 2007-2008. Then, monitor their career trajectories going forward into clerkships, residencies and eventual practice location(s) as being urban, rural, or northern remote. From these trajectories, we wished to determine and evaluate, if any, associations between co- and extra-curricular ORNH activities and current location of practice.

### Data collection

The ORNH maintains extensive database records of participation across its initiatives, though it does not carry responsibility for tracking the trajectories and service paths of physicians. As a result, there is no provision for longitudinal datasets. In 2016, lists of Rural Week participants and their further participation (if any) in other ORNH initiatives were compiled by one of us (DP) from the records of the years 2004-2005 to 2007-2008 inclusive; the first four years that the program was compulsory in Med 1 at the University of Manitoba. Four cohorts (N=368) of Med 1 students (167 female, 201 male) comprised the dataset. Upon initial acquisition of the database, we learned that a significant number of individuals (N=115) remained unaccounted for in terms of present location of practice. In order to make the dataset act closer to a census population, one of us (M) conducted a manual search of publicly-available records including the Canadian Resident Matching Service (CaRMS), the Royal College of Physicians and Surgeons of Canada (RCPSC), the Canadian Institute for Health Information (CIHI), Scott’s Medical Database, and the Canadian Post-MD Education Registry (CAPER) which narrowed this gap substantially. We were able to determine the present location of practice for 232 of the 256 individuals across the four cohorts who were on record as having accessed ORNH programs.

For the purposes of this study, the operational definition of “rural” refers to a physician’s primary practice as located in a community with population less than 20,000 and/or at least 200 km from a Canadian city of population ≥ 200,000; “remote” is defined as a primary practice location in a community with population < 5,000 and ≥ 500 km from a Canadian city of population ≥ 200,000. Since very few of the records indicated current practice as being outside Canada, such records were excluded from the study. The larger proportion of Manitoba physicians classified as in rural practice are located ≤200 km from the major centres of Winnipeg and Brandon.

## Results

**Figure 1:**
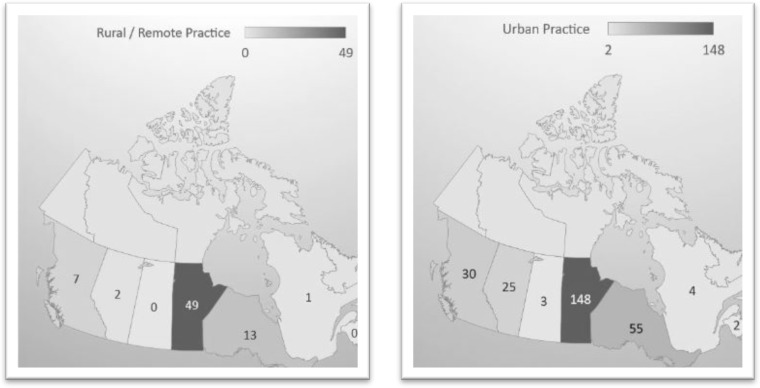
Current geographic distribution of Manitoba-trained physicians from 2004-2007 Rural Week participant cohorts (as at April, 2017; N=74 in rural/remote practice; N=270 in urban). (N = 5 in remote locations are not included in this map)

**Figure 2:**
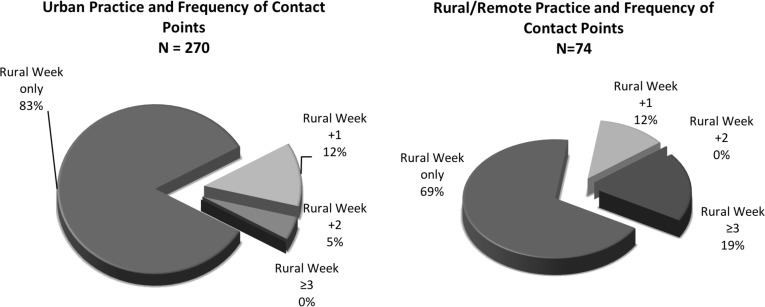
Current location of practice, Manitoba-trained physicians from 2004-2007 Rural Week participant cohorts and frequency of points of contact following Med 1 (as at April, 2017; N=270 urban; N = 74 rural/remote)

Combining all four cohorts, there were 270 in urban practice as of early 2017 and 74 in rural/remote locations of practice. Figures 1 and 2 provide geographic distribution of Manitoba-trained physicians who comprised the 2004-2005 to 2007-2008 cohorts as Med 1 students (updated as of April, 2017; Nunavut, Northwest Territory and Newfoundland & Labrador excepted on maps). The data on physician distribution demonstrates that - for the period 2008 to 2017 - the Province of Manitoba has retained 57.4% of its trained physicians, 14.9% of whom are presently located in rural/remote communities. Among those who are outside Manitoba, a significant proportion are in Canada’s largest urban centres (> 500,000 population) and are engaged in residency, fellowship, or a specialty practice.

As identified earlier, the focus of this study was to examine for associations between matched cohorts of Med 1 students (all of whom participated in a mandatory one-week immersive experience in rural/remote medical practice and the Med 3 rural family medicine clerkship) with these variables; 1) the frequency of points of contact with ORNH programs, and/or; 2) the likelihood of current location of practice to be urban or rural/remote. Figures 2a/b provide complementary illustrations of the relationships among those physicians presently defined as being in urban practice or rural/remote practice and the number of points of contact over the period 2004 – 2016 with ORNH programs:

Fully 83% who are now in urban practice had only the compulsory Rural Week experience in Med 1 and the mandatory clerkship rotation in Med 3. Once the number of further contact points exceeds one intervention, the likelihood of being in urban practice is much reduced such that there were no records of participation in three or more ORNH programs among urban physicians. In contrast, those in rural/remote practice were much more likely to have been participants in additional ORNH contact points. Most visible in the records was that all who had three or more contact points were practicing in rural/remote environs. It is notable, however, that the following association was evident: over half of the physicians in Manitoba in a rural/remote practice location participated only in the mandatory Rural Week (Med 1) and the rural family medicine clerkship rotation (Med 3) as their sole professional learning opportunities oriented explicitly toward rural medical practice.

**Figure 3:**
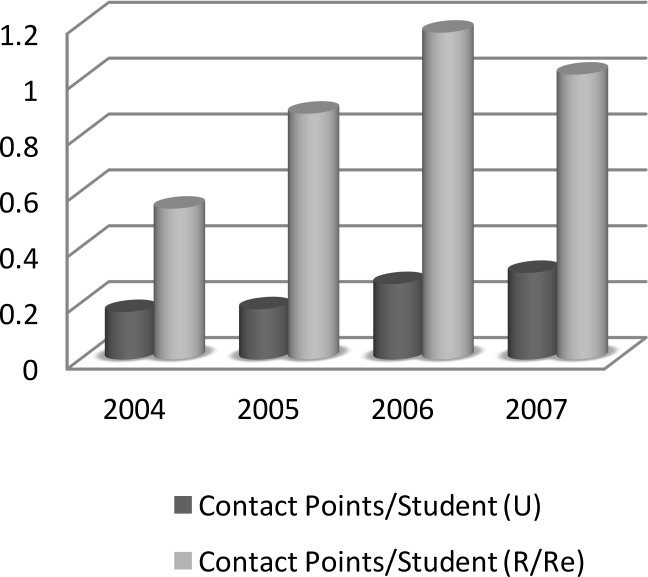
Points of contact per learner and current location of practice (U = urban; R/Re = Rural/Remote)

The data support an association between points of contact per learner over time and location of present practice (Figure 3). Across all four cohorts combined, for those now in urban practice, we observed a mean of 0.233 contact points per individual. Alternatively, if a practitioner is now located in a rural/remote setting there is a mean of 0.903 contact points with ORNH programs per learner. On balance, an association emerges: an individual is four times more likely to be in rural/remote practice by virtue of choosing to participate in one or more of the offerings of the Manitoba Office of Rural and Northern Health beyond the compulsory Rural Week. Fisher exact probabilities for rural/remote versus urban practice (RuRe/U) were as follows: Observed Risk Ratio (RuRe/U) = 2.6757 with lower bound of 1.8513 and upper bound of 3.8671 at confidence interval 0.95; Odds Ratio (RuRe/U) = 4.0244 with lower bound of 2.3009 and upper bound of 7.0388 at confidence interval 0.95. Physicians in rural/remote practice were significantly more likely (*p ≤ 0.05*) to have selected further rural family medicine electives delivered by the ORNH in addition to the mandatory rural rotation experience alone in Med 1 plus the Med 3 clerkship rotation.

## Discussion

This study provides important insights into the structure and functioning of a context-bound and site-specific rural/remote medicine facility and its long-term programming (the ORNH in Manitoba). By virtue of examining multiple cohorts over time and using a “frequency of contact points” determinant among the variables, the study supports these findings. Principal among these is that the association between eventual rural/remote practice is not as strongly associated with the co-curricular ORNH “points of contact” in and of itself. For instance, slightly more than half (55%) of those physicians now in rural/remote locations of practice did not access any of the ORNH programs after Med 1. There is an association among those who had opted for at least one extra-curricular program and strongest if this was three or more “contact points.” Alternatively, when the urban location of practice is examined there are no individuals across the four cohorts who have a record of opting for more than two extra-curricular programs offered be the ORNH. The positive associations, where found, have been identified in terms of odds ratios and relative risk. Factor analysis provides no strong correlates between the choice of certain ORNH electives and retention in rural/remote practice. The voluntary, self-selective and learner-driven nature of the ORNH programming model beyond Med 1 warrants a very cautious approach to data interpretation as is the case in similar matched-cohort analyses in the literature.^4,10-11,20-21,35,40,43,50-52^

Two strengths of this investigation is that the location of almost all the physicians could be determined and the cohort sizes are optimal. Weaknesses of the analysis include no access to data about rural community of origin, location(s) of secondary schooling or other potentially relevant demographic information. This has been demonstrated to be an indicator in student choice to practice rurally.^11^ It is noted that a pre-disposition to choose an orientation to rural practice is a plausible factor in motivating individuals to select these rural-focused contact points. Future analyses would be enhanced if these data were available. The benefit of programming similar to ORNH “points of contact” could then be teased out with respect to one of the other known determinants of rural practice.

A follow-up qualitative study involving a semi-structured interview protocol could uncover the narratives that may contain hidden contributors to the trajectories of Manitoba physicians. It remains important for the ORNH to periodically examine the impact and effectiveness of its “points of contact” to monitor the barriers and the protective factors which apply in particular to the western Canadian context (i.e., Indigenous peoples’ healthcare aspirations, the western Canadian demographic dynamics).^53-55^

### Conclusion

Physicians now in rural/remote practice were significantly more likely to have selected further rural family medicine electives delivered by the ORNH in addition to the mandatory rural rotation experience alone in Med 1 plus the Med 3 clerkship rotation. The study supports, in a retrospective case-study manner, the potential value of continued voluntary interventions designed to support career trajectories in rural/remote practice. Consideration of replicating or hybridizing the ORNH model of professional continuing education elsewhere in Canada is supportable where recruitment and retention remain a continuing challenge.

## Footnotes

a http://www.ornh.mb.ca/rural_week

b http://www.ornh.mb.ca/post_secondary_home_for_the_summer_program

c http://www.umanitoba.ca/medicine/media/Standard_IS-14A_-_Service_Learning_Opportunities.pdf

d http://www.ornh.mb.ca/rural_manitoba_health_mentorship_program

e http://mmsa.online/groups/
